# Editorial: Enhancing sports injury management through medical-engineering innovations

**DOI:** 10.3389/fspor.2026.1872784

**Published:** 2026-05-22

**Authors:** Chen Lu, Chenyu Sun, Penghao Xu, Wei Song, Wencai Liu

**Affiliations:** 1 National Center for Orthopaedics, Shanghai Sixth People’s Hospital Affiliated to Shanghai Jiao Tong University School of Medicine, Shanghai, China; 2 Division of Public Health, Mayo Clinic, Infectious Diseases, and Occupational Medicine, Rochester, MN, United States; 3 Mayo Clinic School of Graduate Medical Education, Mayo Clinic College of Medicine and Science, Rochester, MN, United States; 4 School of Public Health, University of Minnesota-Twin Cities, Minneapolis, MN, United States; 5 Department of Bioinformatics, Georgia Institute of Technology, Atlanta, GA, United States

**Keywords:** artificial intelligence, biomechanics, injury prevention, medical-engineering integration, sports injuries

Sports injuries have become a critical issue affecting public health, the development of competitive sports, and social productivity (1). Conditions such as rotator cuff tears, knee cruciate ligament and meniscal injuries, fragility hip fractures in the elderly, and chronic degenerative lesions of tendons and ligaments not only cause pain and dysfunction but may also lead to reduced athletic performance, diminished quality of life, and even long-term disability. Traditional diagnosis and treatment model heavily rely on physicians’ experience, with notable limitations in precise assessment, individualized treatment, risk prediction, and efficient rehabilitation (2). In recent years, the in-depth interdisciplinary integration of medicine and engineering has driven breakthrough advances across the entire chain of sports injury prevention, diagnosis, treatment and rehabilitation, steering sports medicine from experience-based practice toward precision, intelligence and personalization.

Our special issue collects 24 high-quality research articles covering clinical research, biomechanical simulations, artificial intelligence applications, biomaterial development, rehabilitation engineering, and sports protection. These achievements directly address key clinical challenges and systematically present cutting-edge progress in advanced imaging, intelligent algorithms, digital orthopedics, novel materials and smart rehabilitation equipment, continuously innovating the whole process of sports injury management.

In terms of precise diagnosis and risk assessment, this special issue highlights pivotal progress from morphological judgment to quantitative prediction. Artificial intelligence models based on multimodal imaging and clinical data have significantly improved diagnostic accuracy and prognostic prediction. For instance, KneeXNet integrates graph convolutional networks (GCNs) with a multi-scale feature fusion module to enable automated, high-precision MRI diagnosis of knee injuries (Sun et al.). Yang et al. adopted algorithms such as Least Absolute Shrinkage ang Selection Operator(LASSO) regression and found based on large-sample data that sedentary behavior reduces thyroid hormone sensitivity in males. The Extreme Gradient Boosting(XGBoost) machine learning model demonstrates excellent performance in predicting the risk of avascular necrosis after talus fracture surgery, providing evidence for early individualized intervention in high-risk patients (Zhang et al.). High-precision peripheral quantitative computed tomography (pQCT) dynamically quantifies changes in volumetric bone mineral density after anterior cruciate ligament(ACL) reconstruction, objectively reflects the recovery of joint biomechanics, and underscores the importance of anti-osteoporosis management postoperatively (Yin et al.). A study on the baseline blood flow of six major upper and lower limb arteries in healthy volunteers established normative basal perfusion values for different arteries, offering individualized flow parameters for *ex vivo* perfusion and preservation of amputated limbs (Zhang et al.). These studies have upgraded conventional techniques into a quantifiable, predictable and interpretable precision system, laying a solid foundation for individualized clinical decision-making.

In the field of surgical optimization and biomechanics, multiple studies have advanced the treatment of complex trauma toward precision and stability. For challenging AO/OTA type A2.3 intertrochanteric fractures, the modified cable-plate augmented proximal femora nail antirotaition(PFNA) fixation technique markedly enhances fixation stability, accelerates bone healing, and reduces complication-related risks (Huang et al.). A biomechanical research on periprosthetic clamshell fractures confirmed that cementless long-stem fixation combined with supplemental cerclage wiring can restore joint stability (Zhu et al.). Liu et al. revealed that surgeon handness influences the efficacy and functional outcomes of PFNA surgery, and proposed matching the patient's injured side with the surgeon's dominant hand in surgical scheduling. Xiong et al. conducted finite element analysis on combined ipsilateral femoral neck and femoral shaft fractures, providing quantitative evidence for individualized fixation of high-risk fractures. Finite element modeling also assists surgeons in planning and optimizing meniscal surgery (Yu et al.). Meanwhile, Wang et al. reported a challenging case of total hip arthroplasty for ankylosed hip performed with 3D-printed personalized guides. Intraoperative administration of tranexamic acid (TXA) in total knee arthroplasty can substantially reduce blood loss (Luo et al.). These innovations transform surgery from experience-dependent operations into precise treatment guided by biomechanical principles and empowered by digital technologies.

Regarding biomaterials and tissue regeneration, this special issue illustrates the shift in sports injury repair from passive healing to active regeneration. The ZIF-8-loaded decellularized bioadhesives developed by Jiang et al. can strongly promote tendon-bone healing at the rotator cuff interface. Bai et al. use 3D-bioprinting to create customized grafts, providing promising alternatives for future tendon and ligament repair. A meta-analysis on platelet-rich plasma (PRP) has verified its efficacy in improving physical function and alleviating pain (Fan et al.). Machine learning and nomogram-based models can predict the analgesic effects of postoperative PRP therapy for rotator cuff tears, facilitating the personalized application of PRP (Zhang et al.). Research on the inflammatory mechanisms triggered by metal ions from joint prostheses after arthroplasty provides new insights for mitigating periprosthetic osteolysis and reducing aseptic loosening (Cai et al.). Centered on regenerative medicine, these innovations deliver novel strategies to tackle clinical bottlenecks such as insufficient soft tissue repair strength and high re-tear rates.

In the domain of intelligent rehabilitation and sports protection, medical-engineering integration has propelled rehabilitation from standardized protocols to individualized, precise intervention. A hybrid temporal convolutional networks (TCN) can accurately calculate the internal mechanical loading of the ACL (Li et al.). When combined with resistance braces, it enables intelligent and targeted rehabilitation. Electromyography-driven neuromusculoskeletal (EMG-driven NMSK) models assisted by muscle synergy analysis quantify athletes’ muscle activation levels, serving as a practical tool for training optimization and injury early warning (Chen et al.). A meta-analysis of evidence-based exercise prescriptions for rotator cuff pain confirms that targeted, phased and specific movements can achieve better outcomes with less effort (Wu et al.). Intelligent mouthguards (iMGs) embedded with real-time sensors (Chilmeran et al.) and quantitative video analysis (Aston et al.) are expected to enhance brain protection for athletes in the future. Low-frequency pulsed magnetic fields (PEMF) can significantly improve muscle function (Li et al.). These achievements establish a new intelligent rehabilitation model characterized by real-time monitoring, precise feedback and dynamic adjustment.

Overall, the 24 research papers in this special issue cover a complete research chain, ranging from competitive sports injuries to age-related fragility fractures, from surgical techniques to rehabilitation protocols, and from fundamental mechanisms to clinical translation. They fully embody the interdisciplinary innovation philosophy of clinical demand-oriented, engineering technology-enabled, and functional recovery-targeted. These findings not only address numerous current clinical dilemmas but also lay a solid foundation for intelligent diagnosis, digital orthopedics, regenerative repair and smart rehabilitation. They also demonstrate that medical and engineering innovations will drive earlier, smarter, more precise, more efficient and personalized prevention and treatment in sports medicine. Moving forward, the diagnosis and management of sports injuries will continue to evolve toward full-process digitalization, normalized regenerative medicine, interpretable artificial intelligence, and integrated injury prevention and health maintenance.

We hope readers can gain more than just technological progress, but also a clear perspective on how to solve practical clinical problems and foster broader interdisciplinary collaboration through this issue. Ultimately, we aim to enable all individuals affected by sports injuries—whether elite athletes or the general public—to truly benefit from these innovations, allowing them to exercise safely, recover efficiently, and return to their cherished lives with dignity. This embodies the original mission of sports medicine and fuels our ongoing research and endeavor.
